# Zika virus inhibits cell death by inhibiting the expression of NLRP3 and A20

**DOI:** 10.1128/jvi.01980-24

**Published:** 2025-02-20

**Authors:** Jian Li, Changyang Zhu, Yang Meng, Linliang Zhang, Cong Liu, Yali Qin, Mingzhou Chen

**Affiliations:** 1 State Key Laboratory of Virology and Modern Virology Research Center, College of Life Sciences, Wuhan University 98436 , Wuhan, China; 2 College of Life Sciences, Hubei University 98436 , Wuhan, China; 3 Taikang Center for Life and Medical Sciences, Wuhan University, Wuhan, China; 4 Hubei Jiangxia Laboratory, Wuhan, China; The Ohio State University, Columbus, Ohio, USA

**Keywords:** ZIKV, NLRP3, A20, cell death

## Abstract

Zika virus (ZIKV) is associated with microcephaly in neonates and neurological disorders in adults. Chronic ZIKV infection has been identified in the testes, indicating that the virus can lead to prolonged illness, yet its pathogenesis remains poorly understood. Here, we found that ZIKV infection does not induce significant cell death in mouse macrophages despite the critical role that cell death plays in the antiviral immune response. Furthermore, we discovered that ZIKV infection impairs the activation of the NLPR3-dependent inflammasome and inhibits apoptosis. Consequently, we investigated the regulatory mechanism of the NLRP3 inflammasome and apoptosis in the context of ZIKV infection. Our results revealed significant reductions in the protein expression levels of NLRP3 and A20, attributable to post-transcriptional or translational effects during ZIKV infection. These findings suggest that ZIKV infection may disrupt cell death pathways, leading to its pathogenicity.

**IMPORTANCE:**

Zika virus (ZIKV), first isolated from a nonhuman primate in Africa in 1947, was relatively understudied until 2016. By then, ZIKV had already been reported in more than 20 countries and territories. The infection poses a significant risk, as it is associated with microcephaly in infants and neurological disorders in adults; however, the underlying mechanisms responsible for these severe outcomes remain unclear. In this study, we demonstrate that ZIKV infection significantly reduces the expression of NLRP3 and A20 proteins through post-transcriptional or translational processes, which leads to inhibited cell death. These findings are critical because cell death plays a vital role in the host's antiviral immune response. Our findings highlight how ZIKV infection compromises essential cell death pathways, raising serious concerns about its pathogenesis. A comprehensive understanding of this disruption is vital for developing targeted interventions to mitigate the virus' impact on public health.

## INTRODUCTION

Zika virus (ZIKV) possesses a monopartite, linear, positive-sense RNA genome of approximately 11,000 nucleotides (1). The genome consists of a 5′-untranslated region (UTR) with a standardized cap structure, a single open reading frame (ORF), and a 3′-UTR (1). The ORF encodes a polyprotein that undergoes co-translational processing by both viral and host proteases, yielding 10 mature viral proteins: three structural proteins (C, prM/M, and E) and seven nonstructural (NS) proteins (NS1, NS2A, NS2B, NS3, NS4A, NS4B, and NS5) (1, 2). Initially isolated from a rhesus macaque in Uganda, ZIKV has spread globally in recent years, posing an increasing threat to public health (3, 4). ZIKV infection in pregnant women is particularly concerning, as it is associated with Congenital Zika Syndrome (CZS), including fetal microcephaly (5, 6). Prolonged viremia has also been observed in the semen of infected men (7). Notably, Asian ZIKV strains have been reported to target CD14^+^ blood monocytes, inducing M2-skewed immunosuppression during pregnancy (8), which may be linked to fetal microcephaly. However, the mechanisms underlying fetal microcephaly, immunosuppression, and prolonged infection in ZIKV cases remain largely unknown.

Genetically programmed cell death, including apoptosis, pyroptosis, and necroptosis, is recognized as a crucial mechanism for maintaining homeostasis by eliminating damaged or obsolete cells in both multicellular and unicellular organisms (9). Previous studies have investigated the role of programmed cell death within the innate immune system and during pathogen infections (10–12). Inflammasomes are cytosolic protein complexes that induce lytic and pro-inflammatory pyroptosis, playing a key role in mediating host immune responses and defending against pathogen infections (13). The NLRP3 (NACHT-, leucine-rich-repeat-, and pyrin domain-containing protein 3) inflammasome senses various stimuli and pathogen infections, playing an essential role in maintaining bodily homeostasis (14). Previous studies have reported that the NLRP3 inflammasome is activated by viral infections or their proteins (15–19). A recent study indicated that lyssavirus matrix protein inhibits NLRP3 inflammasome assembly to evade host defenses (20). Similarly, the H240R protein of the African swine fever virus inhibits NLRP3 oligomerization, thereby regulating host immunity (21). However, the mechanism by which host cells induce or inhibit NLRP3 activation during ZIKV infection remains unclear (22–25). We hypothesized that the pathogenic mechanisms of ZIKV may be linked to the response of the NLRP3 inflammasome.

A20, also referred to as TNFAIP3, is an essential anti-inflammatory protein known for its negative regulation of the NF-κB signaling pathway (26). Previous researches have shown that A20 plays a significant role in regulating both cell death and antiviral signaling processes (27, 28). Reduced expression of A20 has been shown to enhance immune response and apoptosis, thereby reducing the production of human respiratory syncytial virus (HRSV) (29). Additionally, A20 interacts with caspase-8 (CASP8) and Fas-associated protein with death domain (FADD) in the context of human T-cell leukemia virus type I (HTLV-I) infected cells, effectively regulating mechanisms of cell death (30). Although existing studies have outlined the regulation of A20 expression in response to ZIKV infection (31, 32), the specific regulatory mechanisms and their functional implications are still not fully understood.

In this study, we investigated how ZIKV infection regulates cell death through cellular morphology and biochemical assays. We found that ZIKV infection does not induce cell death in immortalized bone marrow-derived macrophages (iBMDMs) or bone marrow-derived macrophages (BMDMs). Furthermore, ZIKV infection inhibited drug-induced cell death in both iBMDMs and BMDMs, specifically in response to treatments with lipopolysaccharide (LPS) combined with nigericin (Ni) or with tumor necrosis factor (TNF) combined with cycloheximide (CHX). These findings suggest that ZIKV infection plays a regulatory role in apoptosis and NLRP3 inflammasome-mediated pyroptosis. To explore the mechanism by which ZIKV infection regulates pyroptosis, we reconstituted the NLRP3-EGFP construct in the HeLa cell line. We observed that the level of NLRP3 protein was reduced due to the actions of the ZIKV capsid (C) and/or NS5, but not NS3, likely through post-transcriptional or translational processes. This reduction may contribute to the inhibited activation of the NLRP3 inflammasome. Additionally, we found that the ZIKV C and/or NS5 decreases the levels of the A20 protein, also through post-transcriptional or translational mechanisms rather than through transcriptional regulation or post-translational degradation. In summary, we identified that ZIKV infection inhibits the expression of both NLRP3 and A20 at the protein level, thereby impairing cell death. This impairment may represent a potential mechanism underlying the pathogenicity of ZIKV.

## RESULTS

### ZIKV infection fails to induce cell death in immortalized bone marrow-derived macrophages

**Fig 1:**
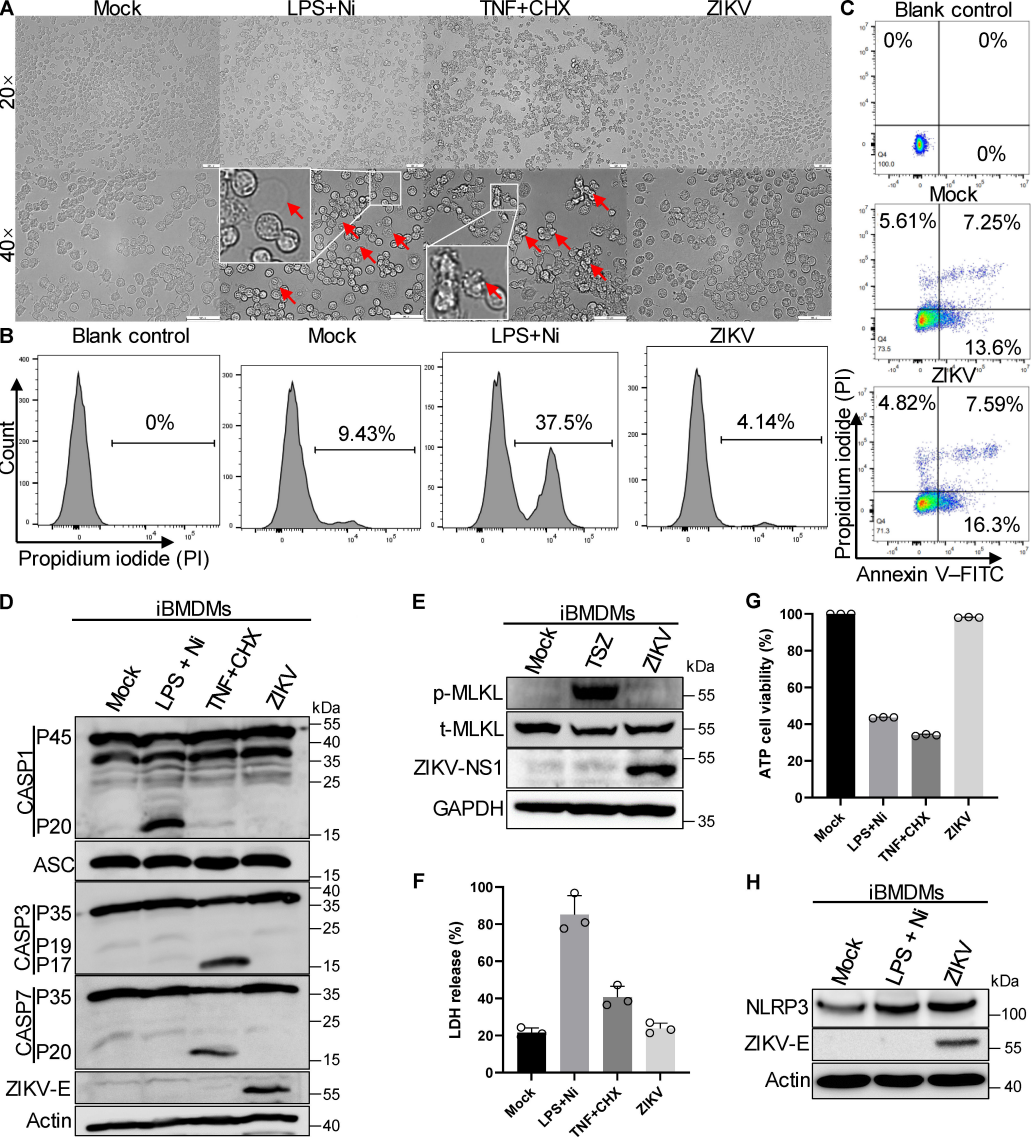
ZIKV infection fails to induce cell death in immortalized bone marrow-derived macrophages. (**A**) Images of iBMDMs after LPS plus nigericin (Ni), TNF plus CHX, or ZIKV infection for 24 h (scale bar, 50 µm). Arrows indicate pyroptotic cells or apoptotic cells. (**B**) Flow cytometry of propidium iodide (PI)-stained iBMDMs. (**C**) Flow cytometry of PI and annexin V-fluorescein isothiocyanate (FITC)-stained iBMDMs. (**D**) Immunoblotting of CASP1, ASC, CASP3, CASP7, and ZIKV E in LPS plus Ni, TNF plus CHX, or ZIKV infection for 24 h. Actin is used as the internal control. (**E**) Immunoblotting of p-MLKL, t-MLKL, and ZIKV NS1 in z-VAD-fmk plus TNF and SM164 or ZIKV infection for 24 h. GAPDH is used as the internal control. (**F**) Comparison of LDH release in iBMDMs with LPS plus Ni, TNF plus CHX, or ZIKV infection for 24 h. (**G**) Comparison of ATP cell viability in iBMDMs with LPS plus Ni, TNF plus CHX, or ZIKV infection for 24 h. (**H**) Immunoblotting of NLRP3 in LPS plus Ni or ZIKV infection for 24 h. Actin is used as the internal control.

Monocytes and macrophages are known to be susceptible to ZIKV and facilitate its infection (33); however, the underlying molecular mechanisms remain poorly understood. To evaluate the impact of ZIKV infection on macrophage cell death, we infected iBMDMs with ZIKV or treated them with LPS plus Ni or TNF plus CHX. We observed no significant differences in cell morphology between the ZIKV-infected group and the mock group. In contrast, treatment with LPS plus Ni and TNF plus CHX induced marked morphological changes associated with pyroptosis and apoptosis, respectively (Fig. 1A). Furthermore, flow cytometry analyses using propidium iodide (PI) staining alone or combined with annexin V staining demonstrated that ZIKV infection did not significantly induce cell death compared to the mock group (Fig. 1B and C). Biochemical analysis of cell death induced by ZIKV infection revealed no cleavage or phosphorylation of key molecules involved in pyroptotic, apoptotic, or necroptotic pathways. In contrast, treatments with LPS plus Ni, TNF plus CHX, and z-VAD-fmk combined with TNF plus SM164 significantly activated the pyroptotic molecule caspase-1 (CASP1), the apoptotic molecules caspase-3 (CASP3) and caspase-7 (CASP7), and the necroptotic molecule mixed-lineage kinase domain-like pseudokinase (MLKL), respectively (Fig. 1D and E). Compared to the mock group, the viability of ZIKV-infected iBMDMs and lactate dehydrogenase (LDH) release remained unchanged after infection. In contrast, treatments involving LPS combined with Ni or TNF with CHX increased LDH release and decreased cell viability (Fig. 1F and G). Previous studies have demonstrated that the induction of NLRP3 expression via NF-κB activation is essential for the activation of the NLRP3 inflammasome (13). In our investigation, we observed an upregulation of NLRP3 expression in ZIKV-infected iBMDMs (Fig. 1H; Fig. S1A), indicating that ZIKV infection activates the NF-κB signaling pathway. Collectively, these findings suggest that ZIKV infection does not significantly induce cell death in iBMDMs but does activate the pro-inflammatory NF-κB pathway.

### ZIKV infection impairs activation of the NLPR3-dependent inflammasome and inhibits apoptosis in iBMDMs and BMDMs

**Fig 2:**
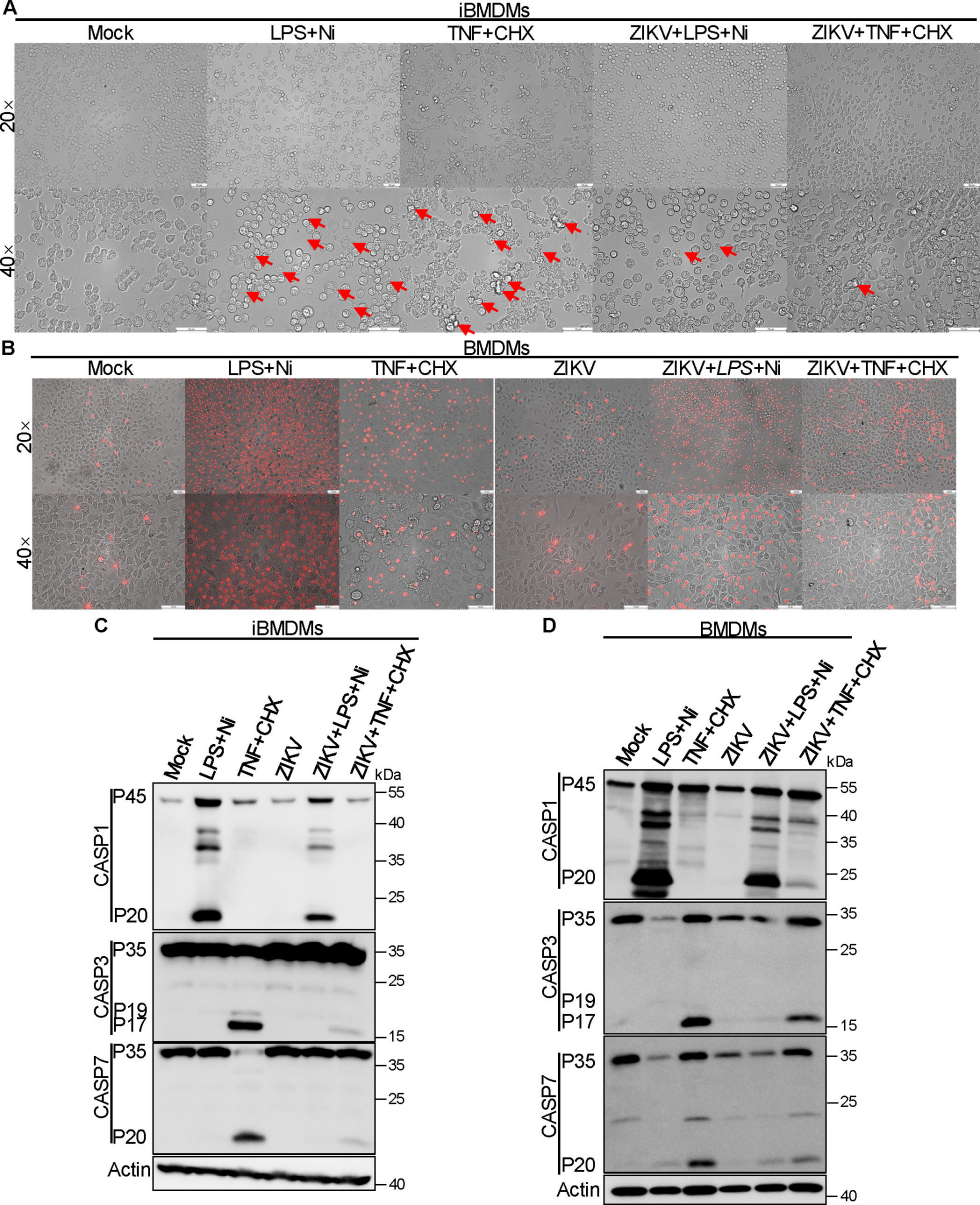
ZIKV infection impairs activation of the NLPR3-dependent inflammasome and inhibits apoptosis in iBMDMs and BMDMs. (**A**) Images of iBMDMs after LPS plus Ni, TNF plus CHX, or ZIKV infection plus the indicated stimulators (scale bar, 50 µm). Arrows indicate pyroptotic cells or apoptotic cells. (**B**) Images of PI-stained BMDMs after LPS plus Ni, TNF plus CHX, ZIKV infection, or ZIKV infection plus the indicated stimulators (scale bar, 50 µm). (**C**) Immunoblotting of CASP1, CASP3, and CASP7 in iBMDMs with LPS plus Ni, TNF plus CHX, ZIKV infection, or ZIKV infection plus the indicated stimulators. Actin is used as the internal control. (**D**) Immunoblotting of CASP1, CASP3, and CASP7 in murine BMDMs with LPS plus Ni, TNF plus CHX, ZIKV infection, or ZIKV infection plus the indicated stimulators. Actin is used as the internal control.

Based on our observations, ZIKV infection does not significantly induce cell death in iBMDMs, prompting us to investigate its effects on the cell death pathway in host cells. Morphological assessments showed decreased cell death in iBMDMs stimulated with LPS plus Ni or TNF plus CHX after ZIKV infection (Fig. 2A). Similarly, ZIKV infection reduced the proportion of propidium iodide (PI)-positive cells in BMDMs treated with the same stimuli (Fig. 2B). Furthermore, ZIKV infection reduced the levels of CASP1-p20 in both iBMDMs and BMDMs treated with LPS plus Ni (Fig. 2C and D), as well as the levels of CASP3-p17 and CASP7-p20 in both cell types treated with TNF plus CHX (Fig. 2C and D). These findings were consistent across both cell types. Additionally, we found that ZIKV infection does not significantly reduce the levels of phosphorylated MLKL (p-MLKL) induced by z-VAD-fmk combined with TNF plus SM164 (TSZ) (Fig. S2), indicating that ZIKV infection does not inhibit necroptosis. Collectively, these results suggest that ZIKV infection may impair the pathways associated with NLPR3-dependent inflammasome activation and apoptosis.

### ZIKV infection inhibits NLRP3 expression independent of autophagy and proteasome pathway

**Fig 3:**
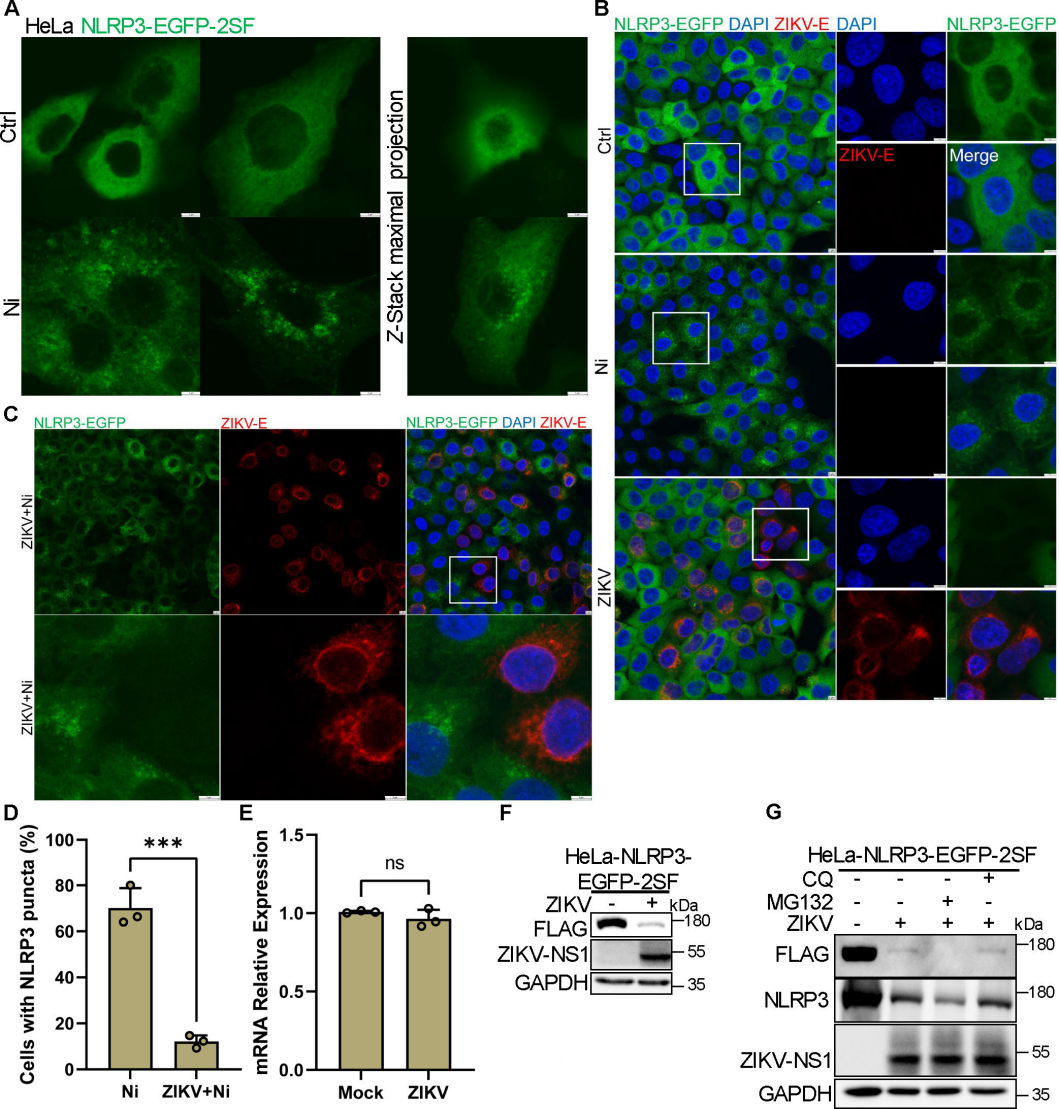
ZIKV infection inhibits NLRP3 expression independent of autophagy and proteasome pathway. (**A**) Confocal images of HeLa cells stably expressing NLRP3-EGFP-2×Strep FLAG (NLRP3-EGFP-2SF) stimulated with 10 µM Ni for 60 min (scale bar, 5 µm). (**B**) Confocal images of HeLa-NLRP3-EGFP-2SF cells stimulated with 10 µM Ni for 60 min or ZIKV infection for 24 h, stained for ZIKV E, and counter-stained with DAPI to visualize nuclei (scale bar, 5 µm). The enlarged field of views is shown for details. (**C**) Confocal images of HeLa-NLRP3-EGFP-2SF cells infected with ZIKV for 24 h plus 10 µM Ni for 60 min and then stained for ZIKV E and counter-stained with DAPI to visualize nuclei (scale bar, 5 µm). The enlarged field of views is shown for details. (**D**) The percentage of cells with NLRP3 puncta was quantified from at least 30 cells stimulated with 10 µM Ni or ZIKV infection plus 10 µM Ni. (**E**) qPCR analysis of *Nlrp3* transcripts in HeLa-NLRP3-EGFP-2SF cells infected with ZIKV. (**F**) Immunoblotting of FLAG and ZIKV NS1 in HeLa-NLRP3-EGFP-2SF cells with ZIKV infection. (**G**) Immunoblotting of FLAG, NLRP3, and ZIKV NS1 in HeLa-NLRP3-EGFP-2SF cells with ZIKV infection combined with CQ or MG132. (**D-E**) Data are presented as the mean ± SD from three independent experiments. Student's *t*-tests were used.

To investigate how ZIKV infection impairs NLRP3 inflammasome activation, we reconstituted NLRP3-EGFP in the HeLa cell line following established protocols (34, 35). Under basal conditions, NLRP3-EGFP was distributed throughout the cytosol of the reconstituted HeLa cells; however, upon treatment with Ni, the protein formed multiple small puncta (Fig. 3A). Previous studies have demonstrated that these puncta represent the activation of NLRP3, confirming the suitability of this reconstitution system for examining the regulation of NLRP3 activation (34). Following ZIKV infection, we observed a significant reduction in EGFP fluorescence intensity in infected cells, as shown by confocal microscopy (Fig. 3B). Immunoblot analysis further confirmed that ZIKV infection significantly decreased the expression levels of NLRP3-EGFP (FLAG-tagged) (Fig. 3F). Moreover, we noted a significant decrease in Ni-induced puncta formation in ZIKV-infected cells (Fig. 3C and D), indicating that ZIKV infection downregulates NLRP3 expression, thereby impairing both NLRP3 puncta formation and inflammasome activation. To investigate the mechanism underlying the downregulation of NLRP3, we conducted quantitative polymerase chain reaction (qPCR) on both ZIKV-infected and mock-infected reconstituted cells. The results revealed no significant differences in transcription levels of reconstituted NLRP3 (Fig. 3E), suggesting that the decreased expression of NLRP3 in infected cells may be attributed to inhibited translation or degradation via autophagy or proteasome pathways.

Given that autophagy and proteasome pathways are known to regulate NLRP3 expression (36–38), we investigated whether these pathways are necessary for the decrease in NLRP3 expression observed following ZIKV infection. However, neither the autophagy inhibitor chloroquine (CQ) nor the proteasome inhibitor MG132 restored NLRP3 expression in ZIKV-infected cells (Fig. 3G). These results indicate that ZIKV infection inhibits NLRP3 expression independent of the autophagy and proteasome pathways, thereby impairing NLRP3 inflammasome activation.

### ZIKV C and NS5, but not NS3, inhibit NLRP3 expression

**Fig 4:**
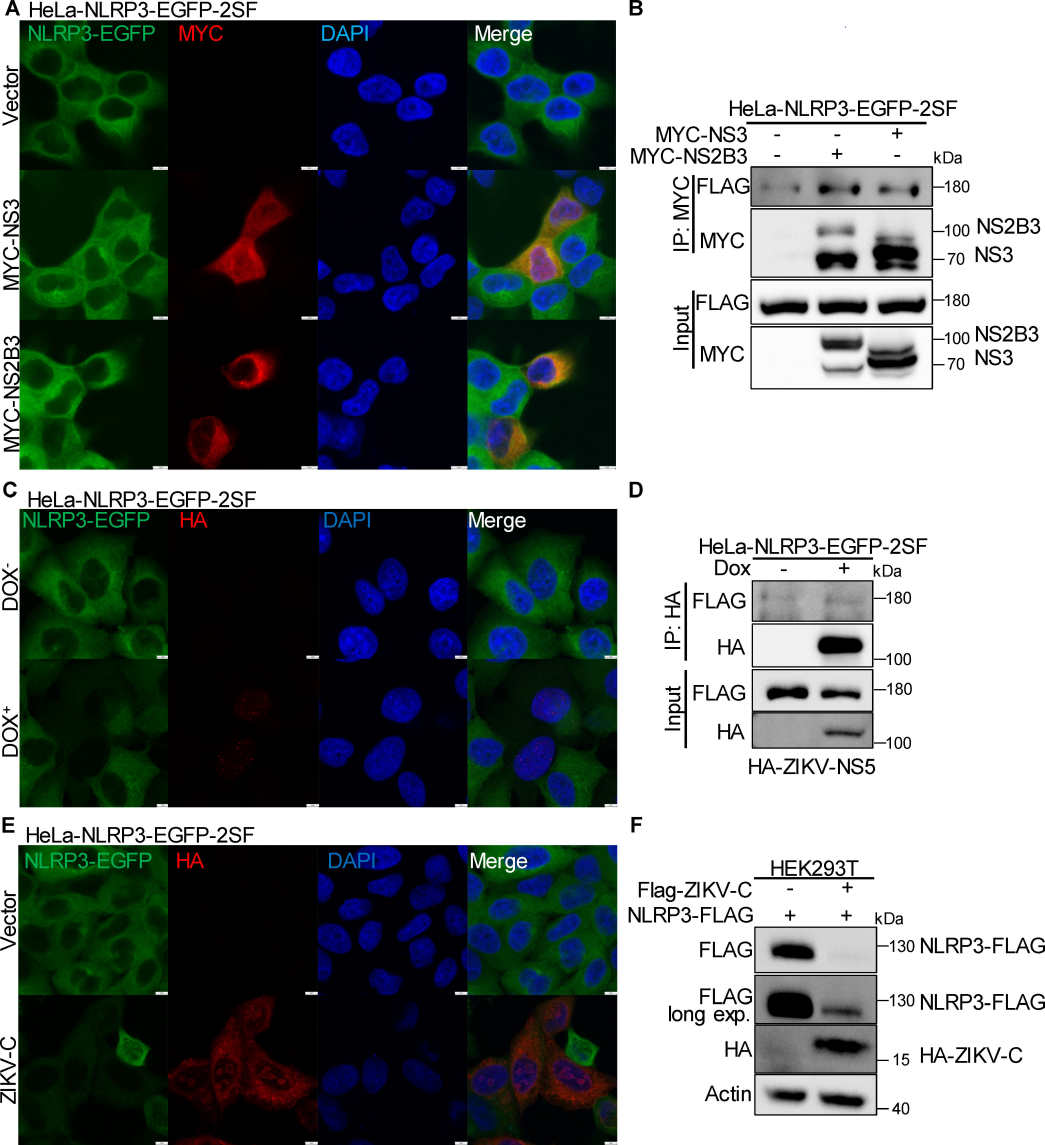
ZIKV C protein and NS5, but not NS3, inhibit NLRP3 expression. (**A**) Confocal images of HeLa-NLRP3-EGFP-2SF cells transfected with ZIKV-NS3, NS2B3, or vector and then stained for antibody against MYC tag and counter-stained with DAPI to visualize nuclei (scale bar, 5 µm). (**B**) Immunoprecipitation assay in HeLa-NLRP3-EGFP-2SF cells transfected with ZIKV-NS3, NS2B3, or vector plasmids. (**C**) Confocal images of HeLa-NLRP3-EGFP-2SF cells expressing Tet-NS5 treated with 1  µg/mL DOX for 48  h then stained for antibody against HA tag and counter-stained with DAPI to visualize nuclei (scale bar, 5 µm). (**D**) Immunoprecipitation assay in HeLa-NLRP3-EGFP-2SF cells expressing Tet vector or Tet-NS5 treated with 1  µg/mL DOX for 48  h. (**E**) Confocal images of HeLa-NLRP3-EGFP-2SF cells transfected with ZIKV-C or vector and then stained for antibody against HA tag and counter-stained with DAPI to visualize nuclei (scale bar, 5 µm). (**F**) Immunoblotting of FLAG, HA, and actin (loading control) in HEK293T cells transfected with ZIKV-C and NLRP3 plasmids.

A previous study suggests that ZIKV NS3 degrades NLRP3 to impair NLRP3 inflammasome activation (23). To determine the effect of NS3 on NLRP3 expression, we transfected NLRP3-EGFP reconstituted HeLa cells with NS3 and found no significant change in NLRP3-EGFP expression (Fig. 4A). Additionally, NS2B has been identified as an essential cofactor of NS3 protease (39, 40). Therefore, we expressed the NS2-B3 fusion protein in NLRP3-EGFP-reconstituted cells, which also showed no significant change in NLRP3-EGFP expression, consistent with the results observed for NS3 alone (Fig. 4A). Immunoprecipitation (IP) assays further confirmed interaction between NLRP3 and both NS3 and NS2-B3; however, NLRP3-EGFP expression remained unchanged, as shown by immunoblotting (Fig. 4B). These results suggest that ZIKV NS3 and NS2-B3 protease do not downregulate NLRP3 expression.

We reconstituted ZIKV NS5 in the HeLa-NLRP3-EGFP cell line using a pLVX vector. After a 48 hour treatment with 1 µg/mL doxycycline (DOX), we confirmed NS5 expression and observed its localization in the nucleus (Fig. 4C), which aligns with previous findings (41). We detected a decrease in NLRP3-EGFP expression in NS5-positive cells, as demonstrated by both immunofluorescence (Fig. 4C) and immunoblotting (Fig. 4D). IP analysis revealed no interaction between NLRP3 and NS5, supporting the observed nuclear localization of NS5 (Fig. 4C and D). Previous studies have suggested that the nuclear localization of NS5 contributes to modulating host immunity by antagonizing type I interferon signaling and promoting a pro-inflammatory response (42, 43). Additionally, as indicated by earlier research (41), we found that ZIKV C was also partially localized in the nucleus (Fig. 4E). Notably, NLRP3-EGFP expression was decreased in C-positive cells, as confirmed by immunofluorescence (Fig. 4E) and immunoblotting (Fig. 4F). In contrast, other ZIKV-encoded proteins did not downregulate NLRP3 expression when co-transfected with the NLRP3 plasmid in HEK293T cells (results not shown). Collectively, these data suggest that ZIKV C and NS5 inhibit NLRP3 expression, likely within the nucleus, while NS3 does not exert this effect.

### ZIKV infection inhibits A20 expression through post-transcriptional mechanisms

**Fig 5:**
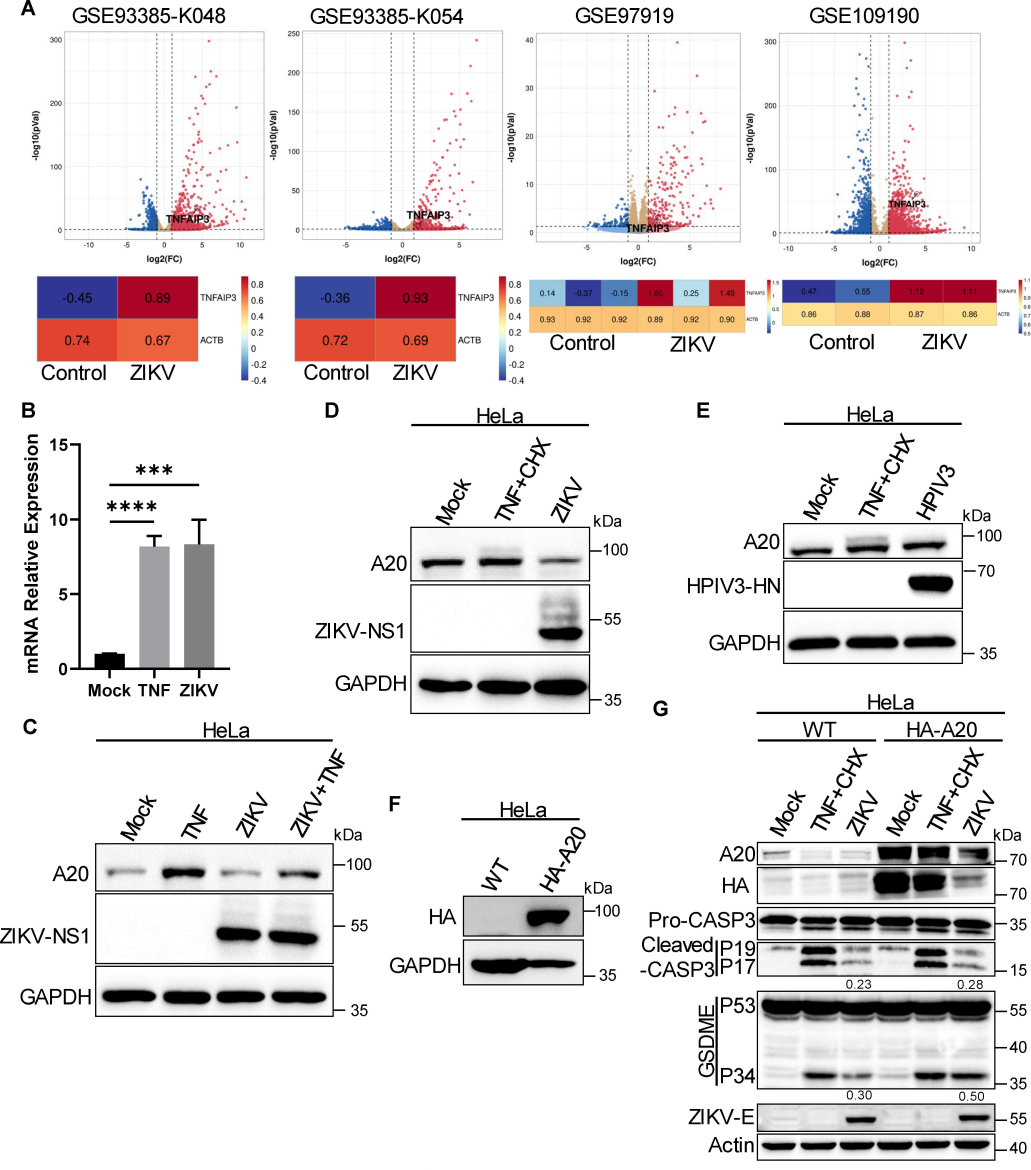
ZIKV infection inhibits A20 expression through post-transcriptional mechanisms. (**A**) RNA sequencing of cells from ZIKV infection compared with mock. Volcano plot of differentially regulated genes, with upregulated genes labeled in red as well as *TNFAIP3*. Heatmaps showing the normalized RNA sequencing values of *TNFAIP3* and *ACTB*. (**B**) qPCR analysis of *TNFAIP3* transcripts in HeLa cells infected with ZIKV or stimulated with TNF. Data are presented as the mean ± SD from three independent experiments. Multiple comparisons of ordinary one-way analysis of variance (ANOVA) were used. (**C**) Immunoblotting of A20, ZIKV-NS1, and GAPDH (loading control) in HeLa cells treated with TNF, ZIKV infection, or ZIKV infection plus TNF. (**D**) Immunoblotting of A20, ZIKV-NS1, and GAPDH (loading control) in HeLa cells treated with TNF plus CHX or ZIKV infection. (**E**) Immunoblotting of A20, HPIV3-HN, and GAPDH (loading control) in HeLa cells treated with TNF plus CHX or HPIV3 infection. (**F**) Immunoblotting of HA and GAPDH (loading control) in HeLa cells stably expressing the HA-A20. (**G**) Immunoblotting of A20, HA, CASP3, GSDME, ZIKV-E protein, and actin (loading control) in HeLa cells or HeLa cells stably expressing the HA-A20 treated with TNF plus CHX or ZIKV infection. For ZIKV infection, the values of CASP3-p17 and GSDME-p34 relative to full-length proteins were presented. The data are representative of three independent experiments.

To explore the mechanisms behind impaired apoptosis in cells infected with the ZIKV, we analyzed publicly available data sets comparing the transcriptomes of ZIKV-infected cells and control cells. This analysis revealed a significant increase in *TNFAIP3* mRNA levels in ZIKV-infected cells (Fig. 5A). Consistent with these transcriptomic findings, qPCR analysis showed that *TNFAIP3* mRNA levels were significantly elevated in both TNF-treated and ZIKV-infected cells compared to mock-treated cells (Fig. 5B). However, immunoblotting showed that A20, the protein encoded by TNFAIP3, was reduced in ZIKV-infected cells relative to the mock group (Fig. 5C and D), suggesting a post-transcriptional or post-translational inhibition of A20 during ZIKV infection. In contrast, infection with human parainfluenza virus type 3 (HPIV3), a negative-sense RNA virus, did not lead to a reduction in A20 expression in HeLa cells (Fig. 5E). In fact, HPIV3 infection was found to induce CASP3 cleavage in iBMDMs and HeLa cells (Fig. S3A through C), indicating that HPIV3 promotes apoptosis in these cell types. Previous studies have reported that A20 inhibits inflammatory NF-κB signaling through a negative feedback loop (27). The NF-κB signaling pathway is well known for facilitating the expression of pro-inflammatory and pro-survival genes (44). Our study also indicated that ZIKV infection activates the phosphorylation of NF-κB p65, resulting in its localization in the nucleus (Fig. S4A and B). This process significantly increased the mRNA levels of pro-survival genes, including *BIRC3*, *BCL-2*, and *MCL-1*, as confirmed by qPCR assays (Fig. S4C). To further explore these processes, we reconstituted the HA-tagged A20 protein in HeLa cells to induce its overexpression (Fig. 5F). Notably, the levels of overexpressed A20 were reduced after ZIKV infection compared to mock-infected cells, suggesting a post-transcriptional inhibition or post-translational degradation of A20 (Fig. 5G). Additionally, we found that overexpression of A20 upregulated cell death, as evidenced by increased CASP3-p17 and GSDME-p34 levels compared to wild-type cells during ZIKV infection (Fig. 5G). Taken together, these results suggest that ZIKV infection downregulates A20 expression, leading to overactivation of the NF-κB pathway, which ultimately promotes cell survival in the context of infection.

### A20 expression is not reduced by post-translational degradation in ZIKV-infected cells

**Fig 6:**
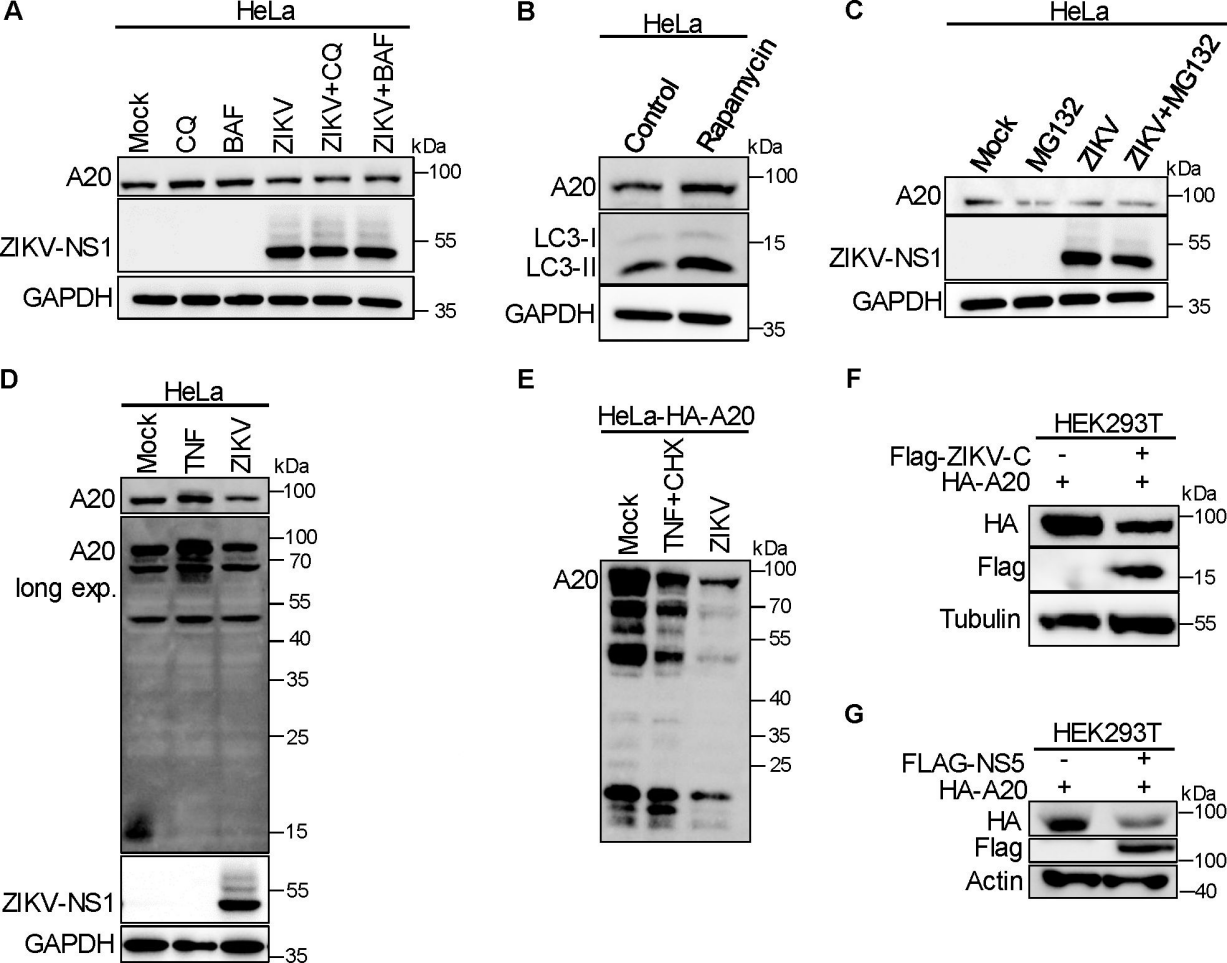
A20 expression is not reduced by post-translational degradation in ZIKV-infected cells. (**A**) Immunoblotting of A20, ZIKV-NS1, and GAPDH (loading control) in HeLa cells infected with ZIKV or ZIKV combined with CQ or BAF. (**B**) Immunoblotting of A20, LC3, and GAPDH (loading control) in HeLa cells treated with rapamycin. (**C**) Immunoblotting of A20, ZIKV-NS1, and GAPDH (loading control) in HeLa cells infected with ZIKV or ZIKV plus MG132. (**D**) Immunoblotting of A20, ZIKV-NS1, and GAPDH (loading control) in HeLa cells treated with TNF or infected with ZIKV. (**E**) Immunoblotting of A20 in HeLa cells stably expressing the HA-A20 treated with TNF plus CHX or infected with ZIKV. (**F**) Immunoblotting of HA, Flag, and tubulin (loading control) in HEK293T cells transfected with HA-A20, Flag-ZIKV-C, or vector plasmids. (**G**) Immunoblotting of HA, Flag, and actin (loading control) in HEK293T cells transfected with HA-A20, Flag-NS5, or vector plasmids.

Previous studies suggest that A20 degradation can occur through several mechanisms, including autophagy (45), the proteasome pathway (46), or cleavage by proteases (47). To investigate the mechanism underlying the reduction of A20 induced by ZIKV infection, we treated ZIKV-infected cells with autophagy inhibitors Bafilomycin A1 (BAF) and CQ. However, we observed no significant increase in A20 expression compared to cells infected with ZIKV alone (Fig. 6A). In contrast, when we induced autophagy in HeLa cells using rapamycin, we noted an upregulation of A20 expression, which starkly contrasted with the reduction observed during ZIKV infection (Fig. 6B). Additionally, we treated ZIKV-infected cells with the proteasome inhibitor MG132 and found no significant increase in A20 expression (Fig. 6C). Previous study has established that A20 cleavage can be mediated by the paracaspase MALT1 (47). In our analysis, we detected no cleavage of A20 in ZIKV-infected cells through immunoblotting, including both wild-type HeLa cells and reconstituted A20 HeLa cells, when compared to the mock and TNF-treated groups (Fig. 6D and E). It is important to note that the samples in Fig. 6E are the same as those for the A20-overexpressing samples in Fig. 5G. Furthermore, immunoblotting results indicated that transfection with the ZIKV C and NS5 significantly reduced A20 expression compared to vector-only transfection (Fig. 6F and G), which corresponds with the reduction mechanism observed for NLRP3. IP analysis revealed no interaction between A20 and ZIKV C or NS5 (Fig. S5A and B). Collectively, these data suggest that the ZIKV-encoding C and NS5 decrease A20 expression during the post-transcriptional phase, rather than through post-translational degradation.

## DISCUSSION

Throughout evolution history, both multicellular organisms and unicellular organisms have developed sophisticated mechanisms to prevent persistent microbial infections by inducing the death in individual cells, thereby maintaining homeostasis within the organism or community (9). It is now widely recognized that cell death plays a crucial role in host defense against viral infections, facilitating the elimination of viruses (9). Different viruses employ distinct strategies to regulate host cell death. Some viruses activate cell death, while others inhibit it to enhance their replication and infection. For example, both norovirus and SARS-CoV-2 have been shown to induce programmed cell death in host cells to complete their life cycle, thereby promoting productive infections (48). In contrast, certain viruses, such as rabies virus (20) and poxviruses (49), suppress host cell death to evade innate immune responses, which fosters viral replication and leads to chronic infections. In this study, we employed an *in vitro* cell culture model to investigate the effects of ZIKV. Our findings indicate that ZIKV not only fails to induce significant programmed cell death but also effectively inhibits exogenous cell death stimuli. We hypothesize that this inhibition may be linked to ZIKV's ability to establish persistent infections and contribute to its pathogenicity.

Apoptosis and pyroptosis are critical processes in the clearance of various viruses and bacteria (50–53). Pyroptosis, a form of inflammatory cell death mediated by gasdermin proteins, results in the formation of pores in the cell membrane (9). The activation of these gasdermin proteins requires upstream inflammasomes activation, which catalyze the maturation of caspase-1. This process triggers the cleavage of GSDMD, initiating pyroptosis. Inflammasomes can recognize pathogen-associated molecular patterns (PAMPs) and danger-associated molecular patterns (DAMPs) either directly or indirectly. Among them, the NLRP3 inflammasome is predominantly expressed in macrophages and other innate immune cells (54). NLRP3 plays a crucial role in orchestrating innate immune responses by responding to a diverse range of PAMPs and DAMPs (55). Notably, ZIKV has been reported to infect human placental and testicular macrophages, establishing long-term infections (33). Therefore, it is essential to understand how macrophages regulate innate immune responses after ZIKV infection.

The NLRP3 inflammasome can be activated by various viral infections, including SARS-CoV-2, influenza virus, and respiratory syncytial virus. This activation leads to the processing of pro-inflammatory cytokines IL-1β and IL-18 by caspase-1, which are then released through GSDMD pores, amplifying antiviral innate immune responses (56). However, activating NLRP3 inflammasome in macrophages during ZIKV infection remains controversial. Wang et al. reported that the ZIKV-encoded NS5 protein can directly interact with NLRP3, activating the inflammasome in THP-1 cells and peripheral blood mononuclear cells (PBMCs) (24). In our study, we observed that NS5 localizes to the nucleus and did not detect any interaction between NS5 and murine NLRP3 in IP assays, which may be attributed to species differences in the cell models used. Consistent with our finding, Gim et al. observed that ZIKV infection impaired NLRP3 inflammasome activation in mouse-derived BMDMs (23). Importantly, we determined that this impairment was not due to NLRP3 cleavage by the NS3, as NLRP3 remained intact even in the presence of the active NS2B-3 protease complex. Immunofluorescence and co-IP assays indicated that both NS3 and NS2B-3 partially colocalized with and interacted with NLRP3, suggesting that NS3 may form a heterologous complex with NLRP3, thereby obstructing NLRP3 oligomerization and subsequent pyroptosis, alone with the relevant immune responses. Both our findings and previous studies confirmed that NS5 localizes to the nucleus, while the C shows partial nuclear localization, specifically within the nucleolus (41). This nuclear localization raises the possibility that ZIKV proteins interfere with the nuclear export or translation of NLRP3 mRNA, as we observed no significant changes in NLRP3 mRNA levels. Notably, nuclear-localized NS5 has been reported to inhibit IRF3-mediated interferon-1 (IFN-1) transcription and decrease SC35 expression (41, 42, 57), supporting the idea that NS5 can broadly impact the expression of host factors and disrupt host antiviral responses. Similarly, the C protein employs post-transcriptional regulatory mechanisms to suppress host antiviral immune responses (58, 59), paralleling our findings regarding the regulation of NLRP3 expression by NS5 and the C protein. However, the precise mechanisms by which NS5 and the C protein modulate host protein mRNA post-transcriptionally remain to be elucidated.

Apoptosis is defined as a non-lytic and immunologically silent form of cell death, which includes two main types: extrinsic apoptosis and mitochondria-mediated intrinsic apoptosis (9). The balance between pro-survival and pro-apoptotic factors is crucial for maintaining normal cellular functions. Under homeostatic conditions, pro-apoptotic signals are suppressed; however, upon stimulation, such as during a viral infection, the downregulation of anti-apoptotic factors allows for the activation of pro-apoptotic pathways. This process is critical for eliminating abnormal cells and pathogens, helping to prevent the spread of infection, and maintaining overall homeostasis. Research suggests that viruses can “hijack” host cell's machinery to manipulate apoptotic pathways (60). By inhibiting apoptosis, viruses can replicate and assemble within infected cells, leading to prolonged infections. The activation of the NF-κB signaling pathway is crucial for promoting the expression of pro-survival mediators to inhibit apoptosis. At the same time, this pathway triggers the expression of negative feedback regulators, such as A20. A20 is an important anti-inflammatory factor that functions through its ubiquitin-editing enzyme activity. This activity effectively suppresses excessive NF-κB activation, helping to prevent the overproduction of both pro-inflammatory and pro-survival mediators (27). Our findings indicate that ZIKV infection disrupts post-transcriptional regulation of A20, resulting in decreased protein levels and sustained activation of the NF-κB pathway, which promotes the production of pro-survival factors. This mechanism could represent a strategy employed by ZIKV to suppress apoptosis and facilitate persistent infection. Similarly to the downregulation of NLRP3 expression, A20 protein levels decreased despite elevated mRNA levels, suggesting that ZIKV may trigger A20 degradation or impede its mRNA translation.

Previous studies have shown that ZIKV can manipulate host protein expression through autophagy and ubiquitin-mediated proteasomal degradation, thereby facilitating immune evasion (61, 62). However, our findings suggest that the decreased levels of the NLRP3 and A20 are not linked to these degradation pathways. We hypothesize that this reduction may result from the inhibition of nuclear export or mRNA translation. Notably, both the ZIKV NS5 and C localize to the nucleus, indicating that ZIKV may disrupt the nuclear export of mRNAs coding for antiviral proteins, thereby suppressing their expression as part of its strategy to modulate host immunity. As a single-stranded, positive-sense RNA virus, ZIKV replicates and assembles in the cytoplasm. However, research has revealed that the nucleus plays a crucial role in the replication of flaviviruses (63). Specifically, the NS5 and C proteins of ZIKV have been found to localize in the nucleus, with the C protein specifically located in the nucleolus (41). Additionally, the NS5 protein of the dengue virus has also been observed to co-localize in the nucleolus (64). These findings suggest that flaviviruses may use their encoded proteins, such as NS5 and C, to manipulate the host's antiviral response, thereby facilitating infection and replication. Studies indicate that the nuclear localization of the NS5 and C proteins is vital for viral infection and replication (65, 66). Notably, inhibiting the entry of NS5 and C into the nucleus can significantly reduce viral infection. However, the detailed function of NS5 and C in the nucleus and nucleolus is still unclear. Further work will be needed to unveil more details of the physiological function of these proteins in the nucleus.

**Fig 7:**
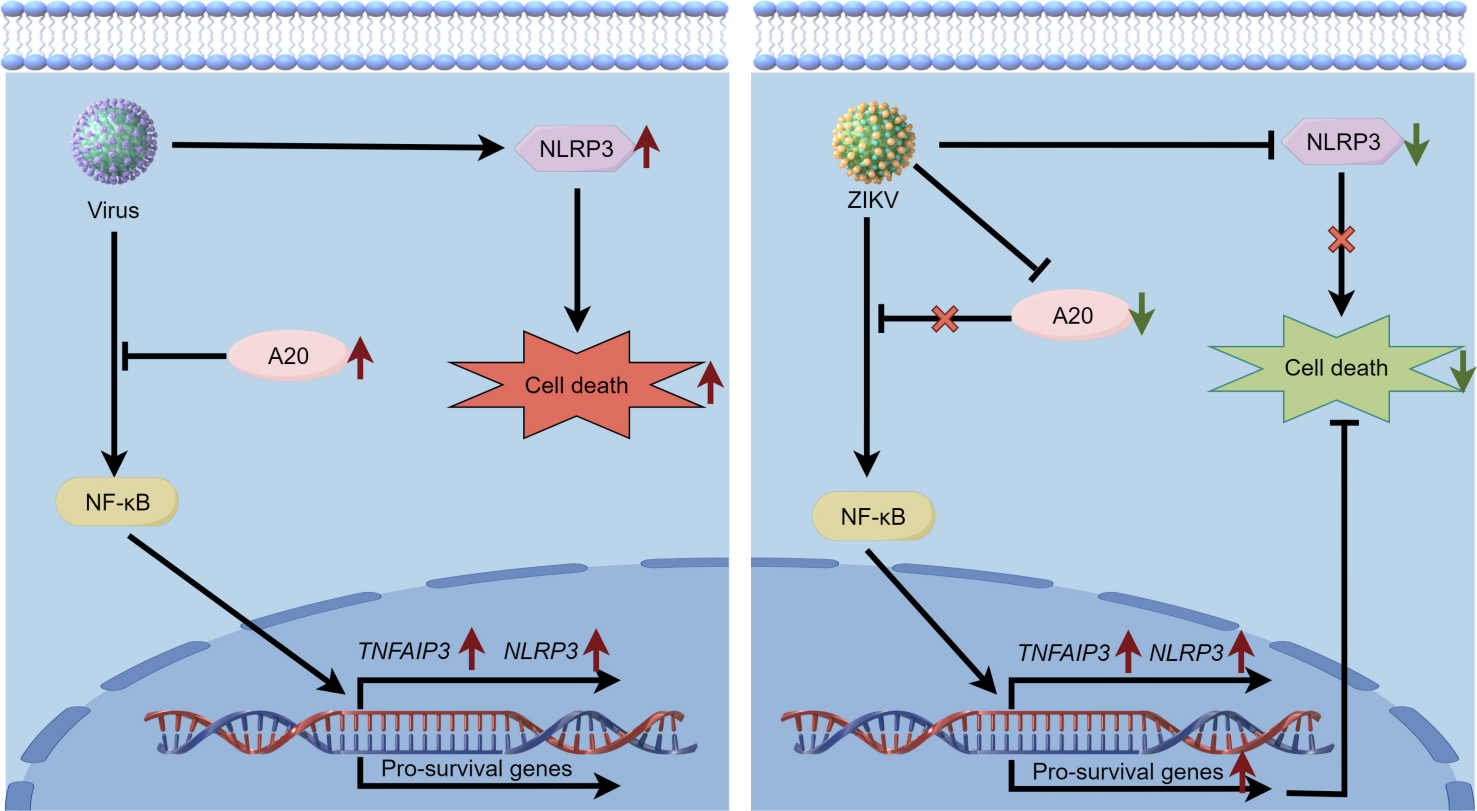
The diagram shows that ZIKV infection inhibits cell death by reducing the expression of NLRP3 and A20 compared to other viruses. The figure was generated using Figdraw.

In conclusion, our study demonstrates that ZIKV infection impedes cell death by downregulating the expression of NLRP3 and A20 (Fig. 7). These findings suggest that the suppression of cell death caused by ZIKV may enable persistent infection and significantly contribute to the virus' pathogenesis.

## MATERIALS AND METHODS

### Cells and viruses

HeLa and HEK293T cell lines were originally obtained from China Center for Type Culture Collection (http://cctcc.whu.edu.cn/). The iBMDMs were kindly provided by Feng Shao (National Institute of Biological Science, Beijing). C6/36 cells were kindly provided by Bo Zhang (Wuhan Institute of Virology, Chinese Academy of Sciences, Wuhan). All the cell lines were cultured in either Dulbecco's modified Eagle's medium (DMEM) (ThermoFisher) or RPMI 1640 (ThermoFisher) supplemented with 10% (vol/vol) fetal bovine serum (WISENT), 1% (vol/vol) penicillin and streptomycin (ThermoFisher), and 1% (vol/vol) GlutaMAX (ThermoFisher).

For the generation of primary BMDMs, cells were isolated from mouse bone marrow and cultured in RPMI 1640 medium (ThermoFisher) containing mCSF-1 (PEPROTECH), supplemented with 10% (vol/vol) fetal bovine serum, penicillin, and streptomycin as mentioned above. These cells were cultured at 37°C in an atmosphere with 5% (vol/vol) CO2. C57BL/6J mice were from GemPharmatech.

ZIKV (SZ01) and HPIV3 were preserved in our laboratory. ZIKV was propagated using C6/36 cells at 27°C as described by Cugola et al. (67). HPIV3 was propagated using LLC-MK2 cells, which were obtained from SUNNCELL, and cultured at 37°C and in a 5% CO2 environment. The titer of ZIKV and HPIV3 was determined by plaque assay as described by Baer et al. (68). All experiments involving ZIKV and HPIV3 infections were conducted in a biosafety level 2 (BSL-2) laboratory.

### Antibodies and reagents

Antibodies against NLRP3 (AG-20B-0014-C100), CASP1 (AG-20B-0042-C100), and ASC (AG-25B-0006-C100) were from AdipoGen. Antibodies against CASP3 (9662), CASP7 (9492), p-MLKL (37333), LC3 (12741), FLAG (14793), and HA (3724) were from Cell Signaling Technology. Antibodies against ZIKV-E (GTX133314) and GAPDH (GTX627408) were from GeneTex. Antibody against Actin (AC026) was from ABclonal Technology. The antibody against MLKL (AP14272b) was from Abgent. Antibodies against ZIKV-NS1 (ab218546) and GSDME (ab215191) were from Abcam. The antibody against A20 (sc-166692) was from Santa Cruz. The antibody against ZIKV-E (BF-1176–46-100UG) for immunofluorescence was from BioFront Technologies. The antibody against MYC (M047-3) was from MBL. Alexa Fluor secondary antibodies (488, 647) (A-11008, A-21235), HRP-conjugated secondary anti-rabbit (31460), and HRP-conjugated secondary anti-mouse (A16066) were from Invitrogen.

LPS (L2880) was from Sigma-Aldrich. TNF-alpha (HY-P7090), cycloheximide (CHX) (HY-12320), Bafilomycin A1 (BAF) (HY-100558), chloroquine (CQ) (HY-17589A), and MG132 (HY-13259) were from MedChemExpress. Nigericin (tlrl-nig) was from InvivoGen. Z-VAD-fmk (S7023), rapamycin (S1039), and SM164 (S7089) were from Selleck.

### Plasmids and cloning

cDNA encoding mouse NLRP3 and human A20 were generated by the standard PCR cloning strategy using iBMDMs and HeLa cells, respectively. The cDNAs were cloned into pCDH or pWPI vector using Hieff Clone Universal II One Step Cloning Kit (Yeasen, 10923ES50) or T4 DNA ligase (ABclonal, RK21501). Plasmids for ZIKV (SZ01) encoding proteins were constructed and preserved in our laboratory. pLVX-TetOne was from Addgene (171123). The primers for PCR amplification are shown in Table S1. All constructed plasmids were determined by DNA sequencing.

### Generation of stable cell lines

HEK293T cells in a 6 cm dish were transfected with 2 µg of transfer plasmid containing the desired gene, 1.6 µg psPAX2 plasmid, and 0.4 µg pMD2.G plasmid using Lipofectamine 2000 reagent according to the manufacturer's instructions. After 48 h of transfection, the lentivirus-containing medium was collected and filtered using a 0.45 µm sterile syringe filter. HeLa cells were infected by adding the specific lentivirus-containing medium to the culture medium with 8 µg/mL polybrene, respectively, followed by 24 h incubation. After 48 h of infection, these infected cells were selected by the 10 µg/mL Blasticidin S (Yeasen, 60218ES50) or puromycin (Yeasen, 60209ES50) containing medium until the wild-type cells died in the control group. The protein expression was examined via Western blot analysis. The HeLa-NLRP3-EGFP-Strep-FLAG stable cell line was selected by cell sorting at SONY MA900 cell sorter, and single clones were also obtained with 96-well plates.

### Viral infection

For *in vitro* infection, iBMDMs and HeLa cells were infected with ZIKV or HPIV3 [multiplicity of infection (MOI), 5] in serum-free media; 1 h after infection, the fresh 10% FBS medium was added to the infected cells. After 24 h infection, the cells were harvested unless otherwise specified.

### Cell stimulation

The iBMDM or BMDM cells were treated with the following cytokines and inhibitor alone or in combinations where indicated: 50 ng/mL LPS, 10 µM nigericin, 20 ng/mL TNF, 10 µg/mL cycloheximide (CHX), 20 µM z-VAD-fmk, 25 µM SM164, 10 µM MG132, 10 nM rapamycin, 10 µM CQ, and 10 nM BAF. To activate the NLRP3 inflammasome, we primed iBMDMs or BMDMs with LPS for 3–4 h and then stimulated them with nigericin for 45 min. For stimulation of extrinsic apoptosis, iBMDMs or BMDMs were treated with TNF plus CHX for 6 h. For stimulation of necroptosis, iBMDMs were treated with z-VAD-fmk for 30 min and then added with TNF and SM164 for 4 h. To stimulate the infected cells, we added the specified stimulators before harvesting. The cells were treated with MG132, CQ, BAF, and rapamycin for 6–8 h.

### Microscopy imaging of cell death

To record cell death morphology, we captured static bright field images of cells infected or stimulated them using a Leica DMi8 microscope. PI staining was also used to evaluate cell death. All image data shown were representative of at least three randomly selected fields.

### Western blot analysis

The harvested cells were lysed in ice-cold RIPA buffer [50 mM Tris (pH 7.4), 150 mM NaCl, 1% Triton X-100, 1% sodium deoxycholate, 0.1% SDS, 1 mM DTT, and protease inhibitor cocktail] for 25 min and then centrifuged at 4°C for 25 min at 20,000 g. The supernatant was subsequently mixed with SDS loading buffer and incubated at 100°C for 10 min. Samples were resolved on 8–15% polyacrylamide gels and transferred onto NC membranes. After being blocked with 5% skin milk for 2 h at room temperature, membranes were incubated with the primary antibodies overnight at 4°C. Subsequently, the blots were washed and then incubated with the horseradish peroxidase (HRP)-conjugated secondary antibodies according to the manufacturer's instructions. Immunoblot images were acquired on the Tanon 5200 automatic chemiluminescence image analysis system. Densitometric measurements of the blots were used with ImageJ software, the measurements were normalized to the loading control, and the experiments were repeated at least three independently.

### Flow cytometry analysis

The treated iBMDMs were harvested and stained with a Dead Cell Apoptosis Kit (Invitrogen, V13242) according to the manufacturer's instructions. The stained cells were determined on a Beckman CytoFlex flow cytometer, and data were analyzed by the FlowJo software.

### Overexpression and immunoprecipitation (IP)

HEK293T cells or HeLa-EGFP cells in six-well plates were transfected with 4–6 µg of interested plasmids and incubated for 24–36 h. Lipofectamine 2000 is commonly used for transfection in immunoprecipitation experiments. Immunoprecipitation was performed as previously described (69). Briefly, cells were harvested and lysed in 0.6 mL ice-cold IP buffer containing 20 mM Tris-HCl (pH 7.5), 150 mM NaCl, 1% Triton X-100, EDTA-free protease inhibitor cocktail, and phosphatase inhibitor cocktail. After lysis on ice for 25 min, the supernatants were harvested by centrifugation at 15,000 g for 25 min. Supernatants for input were set aside 50 µL, and the rest of the supernatants were added 20 µL magnetic or agarose beads conjugated with the indicated antibody. Subsequently, the complex IP samples were incubated overnight at 4°C. The beads were washed four times with IP buffer and boiled in loading buffer and then performed to Western blot analysis.

### Immunofluorescence staining and confocal microscopy

HeLa-NLRP3-EGFP stable cell lines transduced with indicated plasmids were grown on coverslips in 24-well plates. After the corresponding experimental treatment, cells were fixed in 4% paraformaldehyde for 30 min at room temperature and then washed with PBS twice. Subsequently, cells were permeabilized with 0.1% Triton X-100 for 15 min and then washed with PBS twice. Cells were incubated in 3% BSA for 2 h and then incubated with primary antibodies at 4°C for 6–8 h. Cells were washed with PBS and incubated with second antibodies conjugated to a fluorescent probe for 1 h at room temperature, and then a 1 µg/mL DAPI was added for 15 min. Finally, cells were mounted with ProLong Diamond Antifade Mountant on glass slides, and images were obtained using a Leica SP8 confocal microscope with a 63 × 1.4 NA oil objective. Images were analyzed manually using Leica Application Suite X 3.7.4.

### Quantitative RT-PCR analysis

As described by Tu et al. (70), total RNA was extracted from pretreated HeLa cells using TRIzol reagent (Invitrogen, 15596018CN). A reverse transcription procedure was performed by ABScript III RT Master Mix for qPCR with gDNA Remover kit (ABclonal Technology, RK20429) according to the instructions. RT-qPCR was conducted with the CFX384 Real-Time System (Bio-Rad) using 2× Universal SYBR Green Fast qPCR Mix (ABclonal Technology, RK21203). Data were analyzed using the delta-delta Ct method (ddCt), and all results were normalized to *GAPDH* quantified in parallel amplification reactions. The primers used for qPCR are shown in Table S2.

### Cell viability and LDH release assay

Cell death was measured by the LDH assay using CytoTox 96 Non-Radioactive Cytotoxicity Assay kit (Promega, G1780). Cell viability was determined by the CellTiter-Glo Luminescent Cell Viability Assay (Promega, G7570). These experiments were performed as the manufacturer's instructions.

### RNA-seq analysis

To evaluate the role of A20 for ZIKV infection, we performed gene expression analysis of *TNFAIP3* with three data sets deposited in GEO [accession IDs: GSE93385 (71), GSE97919 (72), and GSE109190]. Analysis with GEO2R from NCBI-GEO was used to analyze the gene differential expression.

### Statistical analysis

All experiments were performed as triplicates or more. For all the bar graphs, data were expressed as mean ± SD. Statistical analyses were performed with a standard two-tailed unpaired Student’s *t*-test or multiple comparisons of ordinary one-way ANOVA using GraphPad Prism 9. Quantification of immunofluorescence imaging was performed by counting cells in three individual fields of view for each group. *P* value < 0.05 was considered statistically significant (ns, not significant; *P < 0.05; **P < 0.01; ***P < 0.001; and ****P < 0.0001).

## Data Availability

All plasmids generated in this study are available with a completed Materials Transfer Agreement. Correspondence and requests for materials can be addressed to the corresponding authors.

## SUPPLEMENTAL MATERIAL

Supplemental material jvi.01980-24-s0001.docx Figures S1 to S5; Tables S1 and S2.

ASM does not own the copyrights to Supplemental Material that may be linked to, or accessed through, an article. The authors have granted ASM a non-exclusive, world-wide license to publish the Supplemental Material files. Please contact the corresponding author directly for reuse.
