# Supplementary material for: Zika virus inhibits cell death by inhibiting the expression of NLRP3 and A20
Source: J Virol. 2025 Feb 20;99(3):e01980-24. doi: 10.1128/jvi.01980-24 (PMC11915814; doi:10.1128/jvi.01980-24)
Supplement: Supplemental material — Figures S1 to S5; Tables S1 and S2. [file jvi.01980-24-s0001.docx]

**Supplemental Material**

**Zika virus inhibits cell death by inhibiting the expression of NLRP3 and A20**

Jian Li^1,5^, Changyang Zhu^1,5^, Yang Meng^1^, Linliang Zhang^2^, Cong Liu^1^, Yali Qin^1,2,^*, Mingzhou Chen^1,2,3,4,^*

**Affiliations:**

^1^ State Key Laboratory of Virology and Modern Virology Research Center, College of Life Sciences, Wuhan University, Wuhan, China.

^2^ College of Life Sciences, Hubei University, Wuhan, China.

^3^ Taikang Center for Life and Medical Sciences, Wuhan University, Wuhan, China.

^4^ Hubei Jiangxia Laboratory, Wuhan, China.

^5^ These authors contributed equally.

* Correspondence: yqin@whu.edu.cn (Y.Q.); chenmz@whu.edu.cn (M.C.)

**This file includes:**

Fig. S1-5

Table S1-2


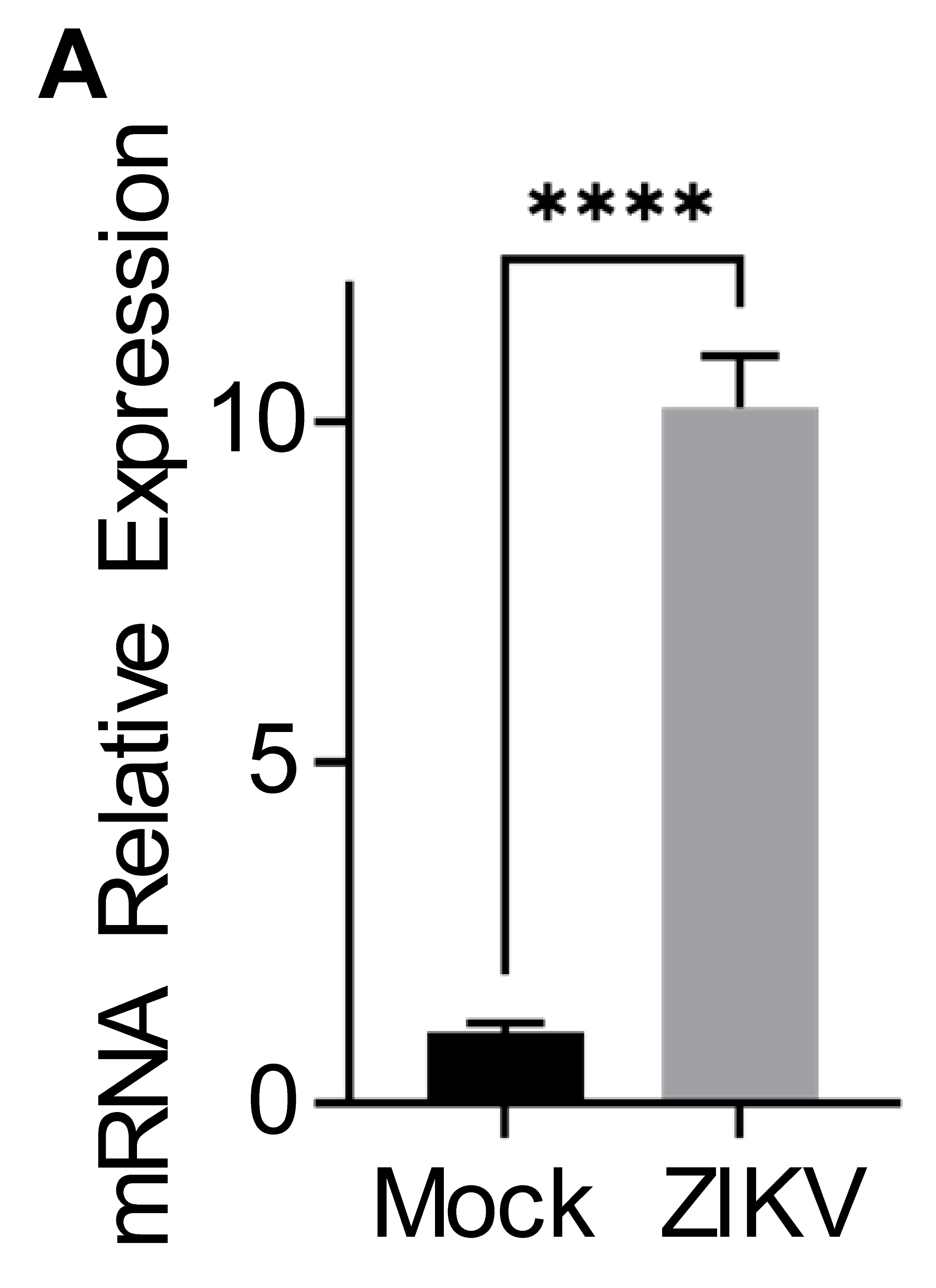


**Fig. S1.** **ZIKV infection leads to an increase in NLRP3 mRNA levels in iBMDMs, related to Fig. 1.**

(A) qPCR analysis of Nlrp3 transcripts in iBMDMs infected with ZIKV.


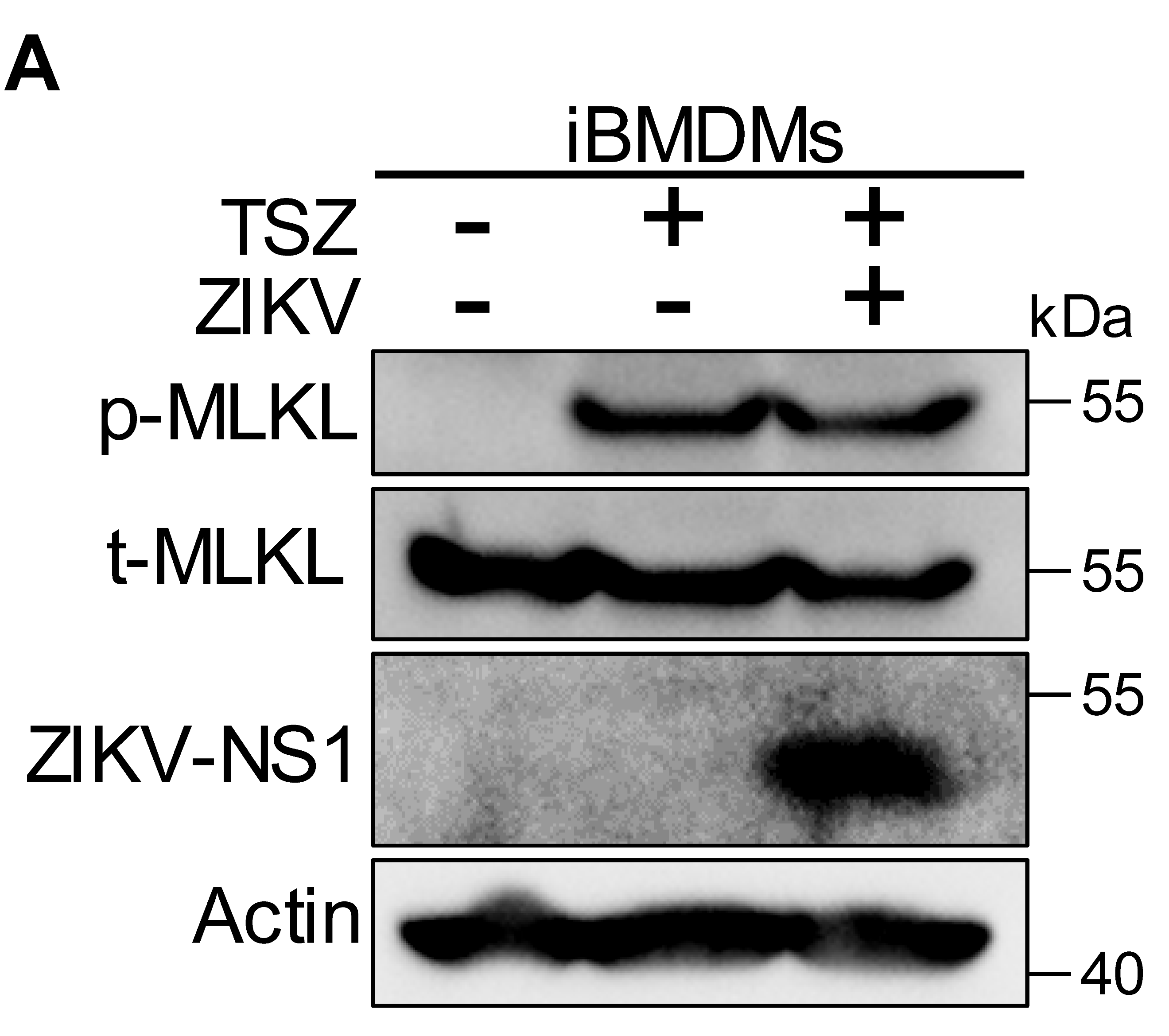


**Fig. S2.** **ZIKV infection does not significantly inhibit necroptosis induced by TSZ, related to Fig. 2.**

(A) iBMDMs were either pretreated with ZIKV infection 24 h prior or not, before the addition of 20 ng/ml TNF (T), 100 nM SM164 (S), and 20 μM z-VAD-fmk (Z) for 3 h. Following this treatment, the levels of phosphorylated MLKL (p-MLKL), total MLKL (t-MLKL), ZIKV NS1, and actin were analyzed through immunoblotting.


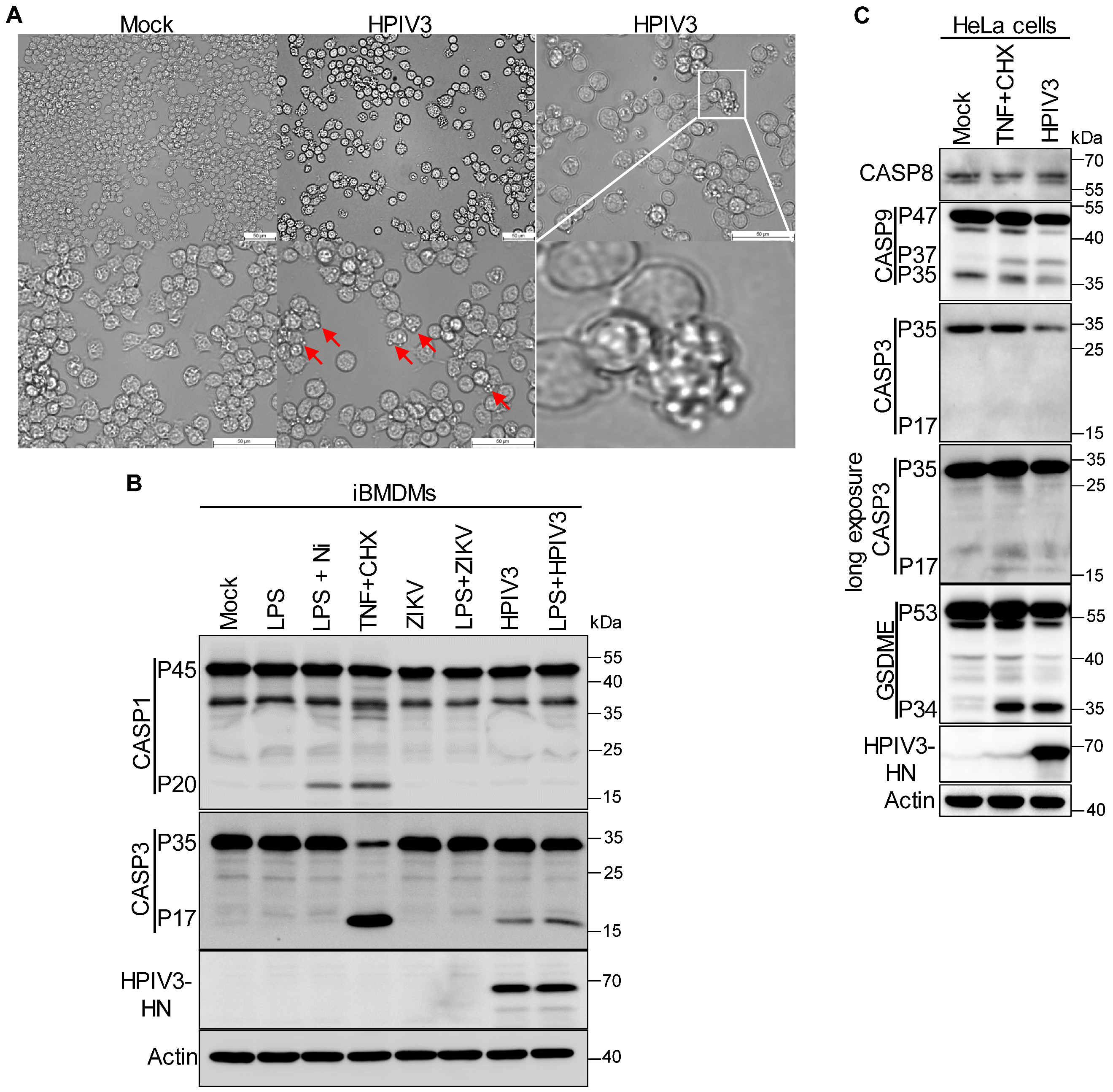


**Fig. S3. HPIV3 infection induces apoptosis in iBMDMs and HeLa cells, related to Fig. 5.**

(A) Images of iBMDM after HPIV3 infection for 24 h (scale bar, 50 um). Arrows indicate apoptotic cells.

(B) Immunoblotting of CASP1, CASP3, and HPIV3-HN protein in mock, LPS, LPS plus Ni, TNF plus CHX, ZIKV infection, ZIKV infection plus LPS, HPIV3 infection, or HPIV3 infection plus LPS. Actin is used as the internal control.

(C) Immunoblotting of CASP8, CASP9, CASP3, GSDME, and HPIV3-HN protein in mock, TNF plus CHX, or HPIV3 infection. Actin is used as the internal control.


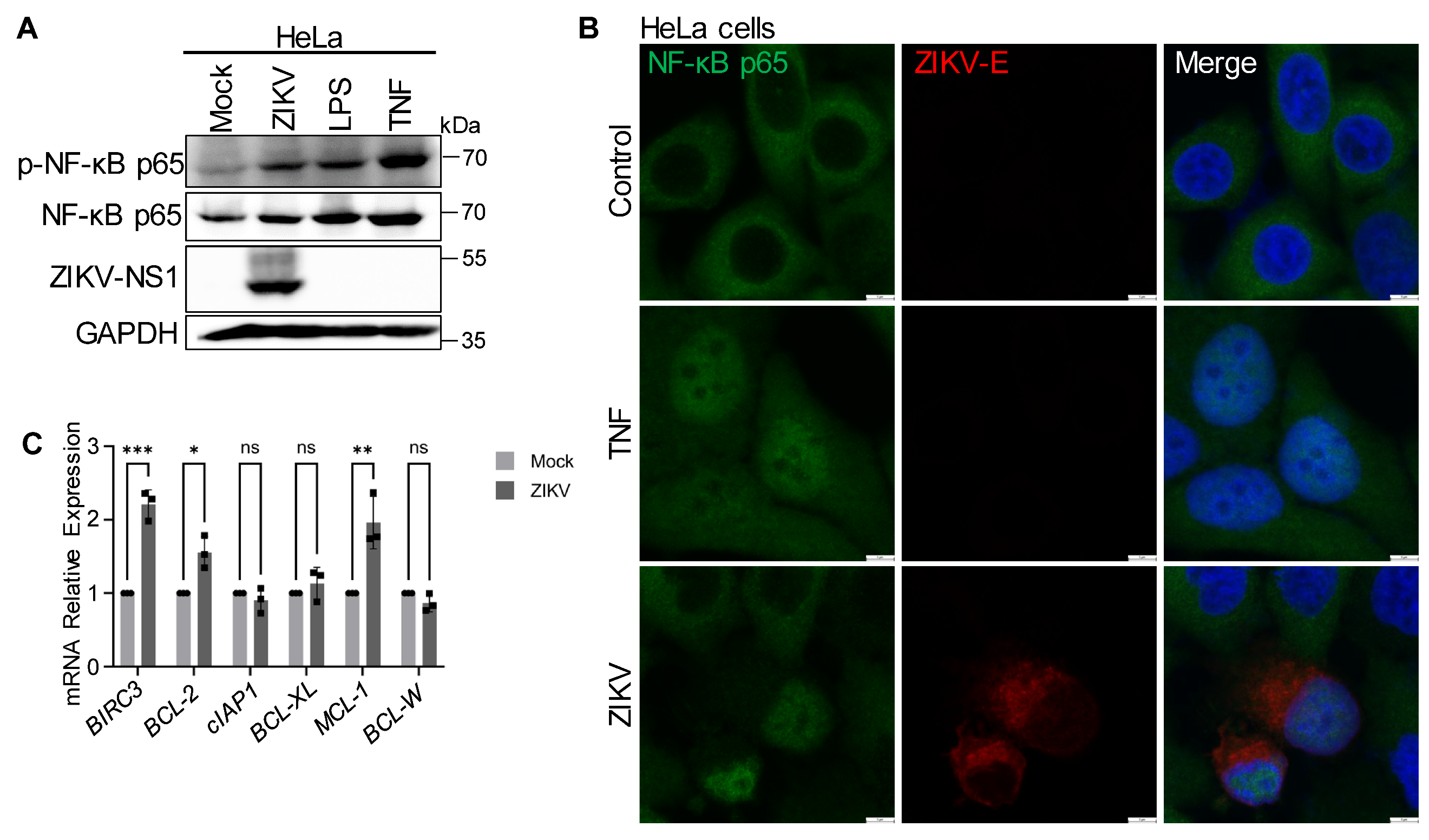


**Fig. S4. ZIKV infection activates the NF-κB signaling pathway and increases the expression of pro-survival genes in HeLa cells, related to Fig. 5.**

(A) Immunoblotting of p-NF-κB p65, NF-κB p65, ZIKV-NS1, and GAPDH in HeLa cells infected with ZIKV, or treated with LPS or TNF. GAPDH is used as the internal control.

(B) Confocal images of HeLa cells stimulated with 20 ng/ml TNF for 2 h or ZIKV infection for 24 h and stained for NF-κB p65, ZIKV-E protein, and conter-stained with DAPI to visualize nuclei (scale bar, 5 μm).

(C) qPCR analysis of *BIRC3*, *BCL-2*, *cIAP1*, *BCL-XL*, *MCL-1*, and *BCL-W* transcripts in HeLa cells from the ZIKV infection group or mock group.


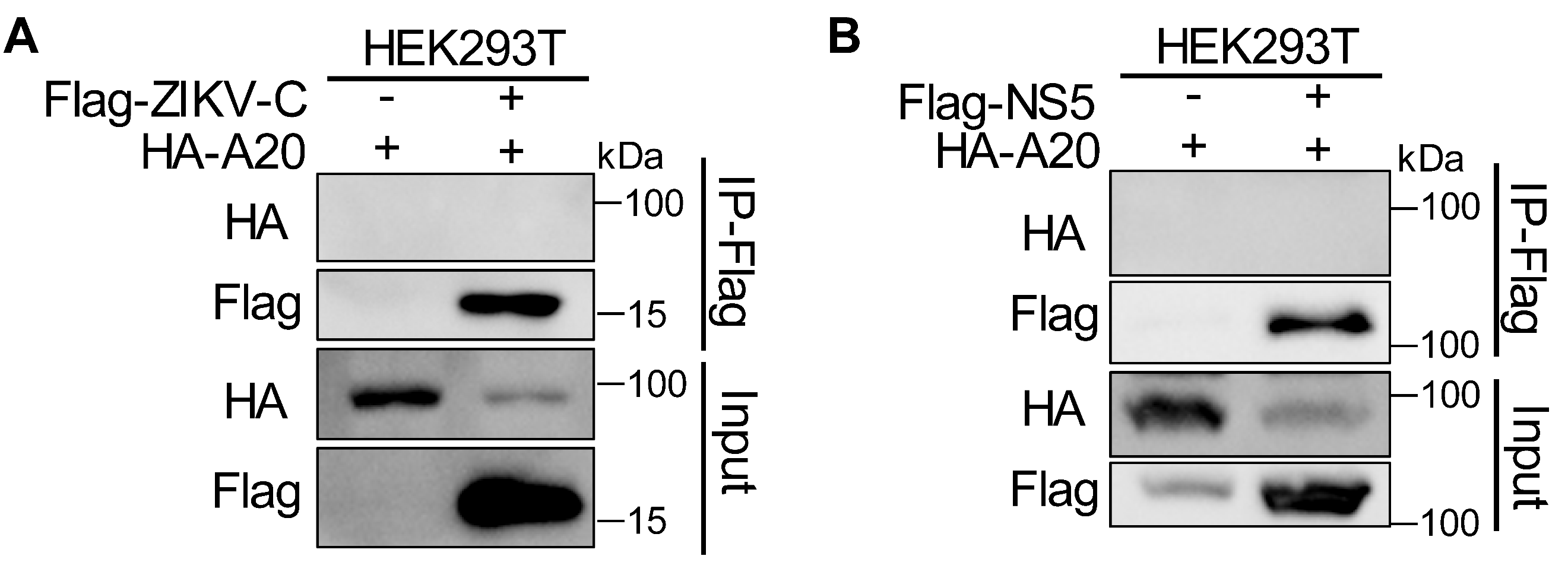


**Fig. S5.** **A20 does not interact with ZIKV C or NS5 in HEK293T cells, related to Fig. 6.**

(A) Immunoprecipitation assay in HEK293T cells transfected with HA-tagged A20, Flag-tagged ZIKV C, or vector plasmids.

(B) Immunoprecipitation assay in HEK293T cells transfected with HA-tagged A20, Flag-tagged ZIKV NS5, or vector plasmids.

**Table S1 Primers for PCR amplification**

| Plasmids |  | Primers |
| --- | --- | --- |
| pCDH-NLRP3-EGFP | F1 | atctcgagctcaagcttcgaattcATGACGAGTGTCCGTTGCAAG |
|  | R1 | cagctcctcgcccttgctcaccatCCAGGAAATCTCGAAGACTATAGTCAGC |
|  | F2 | atggtgagcaagggcgagg |
|  | R2 | tcgaactgagggtggctccaggatcccttgtacagctcgtccatgccg |
| pCDH-NLRP3-FLAG | F | atctcgagctcaagcttcgaattcATGACGAGTGTCCGTTGCAAG |
|  | R | tcgaactgagggtggctccaggatccCCAGGAAATCTCGAAGACTATAGTCAGC |
| pWPI-HA-A20 | F | cacGAATTCatggctgaacaagtcctt |
|  | R | CACtctagagccatacatctgcttgaa |
| pLVX-HA-ZIKV-NS5 | F | ACGATGTTCCAGATTACGCTaccggtGGGGGTGGAACAGGAGAGAC |
|  | R | gctcgcaggggaggtggtctggatccctaCAGCACTCCAGGTGTAGACCC |

**Table S2 Primers for qPCR amplification**

| Gene | Primer F | Primer R |
| --- | --- | --- |
| m*Nlrp3* | ATTACCCGCCCGAGAAAGG | TCGCAGCAAAGATCCACACAG |
| h*TNFAIP3* | cgcaaagttggatgaagc | tccatgagagaaagctggg |
| h*BIRC3* | AGACAGAGTGGCTTGCTTTGC | GCATTATCCTTCGGTTCCCAAT |
| h*BCL-2* | CATGTGTGTGGAGAGCGTCAA | TCATCCACAGGGCGATGTT |
| h*cIAP1* | TGCCTGTGGTGGGAAGCT | CCGGTGTTCTGACATAGCATCA |
| h*BCL-XL* | GCCACTTACCTGAATGACC | TGAGCCCAGCAGAACC |
| h*MCL-1* | CGACGGCGTAACAAACT | GGAAGAACTCCACAAACCC |
| h*BCL-W* | GAGCCATATAGTTCCTTGGGA | TAGAATAAGTGGGGAGTGGGA |
| h*GAPDH* | GCCAAGGCTGTGGGCAAGG | GGAGGAGTGGGTGTCGCTG |
